# Evaluating the effect of annotation size on measures of semantic similarity

**DOI:** 10.1186/s13326-017-0119-z

**Published:** 2017-02-13

**Authors:** Maxat Kulmanov, Robert Hoehndorf

**Affiliations:** 1 Computational Bioscience Research Center, King Abdullah University of Science and Technology, Thuwal, 23955-6900 Saudi Arabia; 2 Computer, Electrical and Mathematical Sciences and Engineering Division, King Abdullah University of Science and Technology, Thuwal, 23955-6900 Saudi Arabia

**Keywords:** Semantic similarity, Ontology, Gene ontology

## Abstract

**Background:**

Ontologies are widely used as metadata in biological and biomedical datasets. Measures of semantic similarity utilize ontologies to determine how similar two entities annotated with classes from ontologies are, and semantic similarity is increasingly applied in applications ranging from diagnosis of disease to investigation in gene networks and functions of gene products.

**Results:**

Here, we analyze a large number of semantic similarity measures and the sensitivity of similarity values to the number of annotations of entities, difference in annotation size and to the depth or specificity of annotation classes. We find that most similarity measures are sensitive to the number of annotations of entities, difference in annotation size as well as to the depth of annotation classes; well-studied and richly annotated entities will usually show higher similarity than entities with only few annotations even in the absence of any biological relation.

**Conclusions:**

Our findings may have significant impact on the interpretation of results that rely on measures of semantic similarity, and we demonstrate how the sensitivity to annotation size can lead to a bias when using semantic similarity to predict protein-protein interactions.

**Electronic supplementary material:**

The online version of this article (doi:10.1186/s13326-017-0119-z) contains supplementary material, which is available to authorized users.

## Background

Semantic similarity measures are widely used for datamining in biology and biomedicine to compare entities or groups of entities in ontologies [1, 2], and a large number of similarity measures has been developed [3]. The similarity measures are based on information contained in ontologies combined with statistical properties of a corpus that is analyzed [1]. There are a variety of uses for semantic similarity measures in bioinformatics, including classification of chemicals [4], identifying interacting proteins [5], finding candidate genes for a disease [6], or diagnosing patients [7].

With the increasing use of semantic similarity measures in biology, and the large number of measures that have been developed, it is important to identify a method to select an adequate similarity measure for a particular purpose. In the past, several studies have been performed that evaluate semantic similarity measures with respect to their performance on a particular task such as predicting protein-protein interactions through measures of function similarity [8–10]. While such studies can provide insights into the performance of semantic similarity measures for particular use cases, they do not serve to identify the *general* properties of a similarity measure, and the dataset to be analyzed, based on which the suitability of a semantic similarity measure can be determined. Specifically, when using semantic measures, it is often useful to know how the annotation size of an entity affects the resulting similarity, in particular when the corpus to which the similarity measure is applied has a high variance in the number of annotations. For example, some semantic similarity measures may always result in higher similarity values when the entities that are compared have more annotations and may therefore be more suitable to compare entities with the same number of annotations. Furthermore, the difference in annotation size can have a significant effect on the similarity measure so that comparing entities with the same number of annotations may always lead to higher (or lower) similarity values than comparing entities with a different number in annotations.

Here, we investigate features of a corpus such as the number of annotations to an entity and the variance (or difference) in annotation size on the similarity measures using a large number of similarity measures implemented in the Semantic Measures Library (SML) [11]. We find that different semantic similarity measures respond differently to annotation size, leading to higher or lower semantic similarity values with increasing number of annotations. Furthermore, the difference in the number of annotations affects the similarity values as well. Our results have an impact on the interpretation of studies that use semantic similarity measures, and we demonstrate that some biological results may be biased due to the choice of the similarity measure. In particular, we show that the application of semantic similarity measures for predicting protein-protein interactions can result in a bias, similarly to other ‘guilt-by-association’ approaches [12], in which the sensitivity of the similarity measure to the annotation size confirms a bias present in protein-protein interaction networks so that well-connected and well-annotated proteins have, on average, a higher similarity by chance than proteins that are less well studied.

## Methods

### Generation of test data

We perform all our experiments using the Gene Ontology (GO) [13], downloaded on 22 December 2015 from http://geneontology.org/page/download-ontology and Human Phenotype Ontology (HPO) [14], download on 1 April 2016 from http://human-phenotype-ontology.github.io/downloads.html in OBO Flatfile Format. The version of GO we use consists of 44,048 classes (of which 1941 are obsolete) and HPO consists of 11,785 classes (of which 112 are obsolete). We run our experiments on several different sets of entities annotated with different number of GO or HPO classes and one set of entities annotated with GO classes from specific depth of the graph structure. The first set contains 5500 entities and we randomly annotated 100 entities each with 1,2,…,54,55 GO classes. We generate our second set of entities annotated with HPO classes in the same fashion. The third set is a set of manually curated gene annotations from the yeast genome database file (gene_associations.sgd.gz) downloaded on 26 March 2016 from http://www.yeastgenome.org/download-data/curation. The dataset consists of 6108 genes with annotations sizes varying from 1 to 55, and each group of the same size contains a different number of gene products. We ignore annotations with GO evidence code ND (No Data). The fourth set contains 1700 entities which is composed of 17 groups. Each group have 100 randomly annotated entities with GO classes from the same depth of the ontology graph structure.

### Computing semantic similarity

After the random annotations were assigned to the entities, we computed the semantic similarity between each pair of entities using a large set of semantic similarity measures. We include both groupwise measures and pairwise measures with different strategies of combining them [1]. Groupwise similarity measures determine similarity directly for two sets of classes. On the other hand, indirect similarity measures first compute the pairwise similarities for all pairs of nodes and then apply a strategy for computing the overall similarity. Strategies for the latter include computing the mean of all pairwise similarities, computing the Best Match Average, and others [1].

Furthermore, most semantic similarity measures rely on assigning a weight to each class in the ontology that measures the specificity of that class. We performed our experiments using an intrinsic information content measure (i.e., a measure that relies only on the structure of the ontology, not on the distribution of annotations) introduced by [15].

The semantic similarity measures we evaluated include the complete set of measures available in the Semantic Measures Library (SML) [11], and the full set of measures can be found at http://www.semantic-measures-library.org. The SML reduces an ontology to a graph structure in which nodes represent classes and edges in the graph represent axioms that hold between these classes [16, 17]. The similarity measures are then defined either between nodes of this graph or between subgraphs.

The raw data and evaluation results for all similarity measures are available as Additional file 1: Table S1. The source code for all experiments is available on GitHub at https://github.com/bio-ontology-research-group/pgsim.

### Measuring correlation

In order to measure the sensitivity of the similarity measures to the number of annotations we calculated Spearman and Pearson correlation coefficients between set of annotations sizes and the set of average similarity of one size group to all the others. In other words, we first computed the average similarities for each entity in a group with fixed annotation size and computed the average similarity to all entities in our corpus. For calculating the correlation coefficients we used SciPy library [18].

### Protein-protein interactions

We evaluate our results using protein-protein interaction data from BioGRID [19] for yeast, downloaded on 26 March 2016 from http://downloads.yeastgenome.org/curation/literature/interaction_data.tab. The file contains 340,350 interactions for 9868 unique genes. We filtered these interactions using the set of 6108 genes from the yeast genome database and our final interaction dataset includes 224,997 interactions with 5804 unique genes. Then we compute similarities between each pair of genes using simGIC measure [1] and Resnik’s similarity measure [20] combined with Average and Best Match Average (BMA) strategies and generate similarity matrices. Additionally, we create a dataset with random GO annotations for the same number of genes, and the same number of annotations for each gene. We also generate the similarity matrices for this set using the same similarity measures. To evaluate our results, we use the similarity values as a prediction score, and compute the receiver operating characteristic (ROC) curves (i.e., a plot of true positive rate as function of false positive rate) [21] for each similarity measure by treating pairs of genes that have a known PPI as positive and all other pairs of proteins as negatives.

In order to determine if our results are valid for protein-protein interaction data from other organisms, we perform a similar evaluation with mouse and human interactions. We downloaded manually curated gene function annotations from http://www.geneontology.org/gene-associations/ for mouse (gene_associations.mgi.gz) and human (gene_associations.goa_human.gz) on 12 November 2016. The mouse annotations contain 19,256 genes with annotations size varying from 1 to 252 and human annotations contain 19,256 genes with annotations size varying from 1 to 213. We generate random annotations with the same annotations sizes for both datasets and compute similarity values using Resnik’s similarity measure combined with BMA strategy. For predicting protein-protein interactions we use BioGRID interactions downloaded on 16 November 2016 from https://thebiogrid.org/download.php. There are 38,513 gene interactions for mouse and 329,833 interactions for human.

### Gene-Disease associations

To evaluate our results with differnt ontologies, we aim to predict gene–disease associations using phenotypic similarity between genes and diseases. We use mouse phenotype annotations and mouse gene–disease associations downloaded from http://www.informatics.jax.org/downloads/reports/index.html(MGI_PhenoGenoMP.rpt and MGI_Geno_Disease.rpt). The dataset contains 18,378 genes annotated with Mammalian Phenotype Ontology (MPO) [22] classes with size varying from 1 to 1671, and 1424 of genes have 1770 associations with 1302 Mendelian diseases. We downloaded Mendelian disease phenotype annotations from http://compbio.charite.de/jenkins/job/hpo.annotations.monthly/lastStableBuild/ and generated random annotations with the same sizes for both gene and disease annotation datasets. We computed similarity of each gene to each disease by computing the Resnik’s similarity measure combined with BMA strategy between sets of MPO terms and HPO terms based on PhenomeNET Ontology [6]. Using this similarity value as a prediction score we computed ROC curves for real and random annotations.

## Results and discussion

Our aim is to test three main hypothesis. First, we evaluate whether the annotation size has an effect on similarity measures, and quantify that effect using measures of correlation and statistics. We further evaluate whether annotation size has an effect on the variance of similarity values. Second, we evaluate whether the difference in the number of annotations between the entities that are compared has an effect on the similarity measure, and quantify the effects through measures of correlation. Third, we evaluate whether the depth of the annotation classes has an effect on similarity measures. Finally, we classify semantic similarity measures in different categories based on how they behave with respect to annotation size, differences in annotation size and depth of annotation classes, using the correlation coefficients between similarity value.

**Fig. 1:**
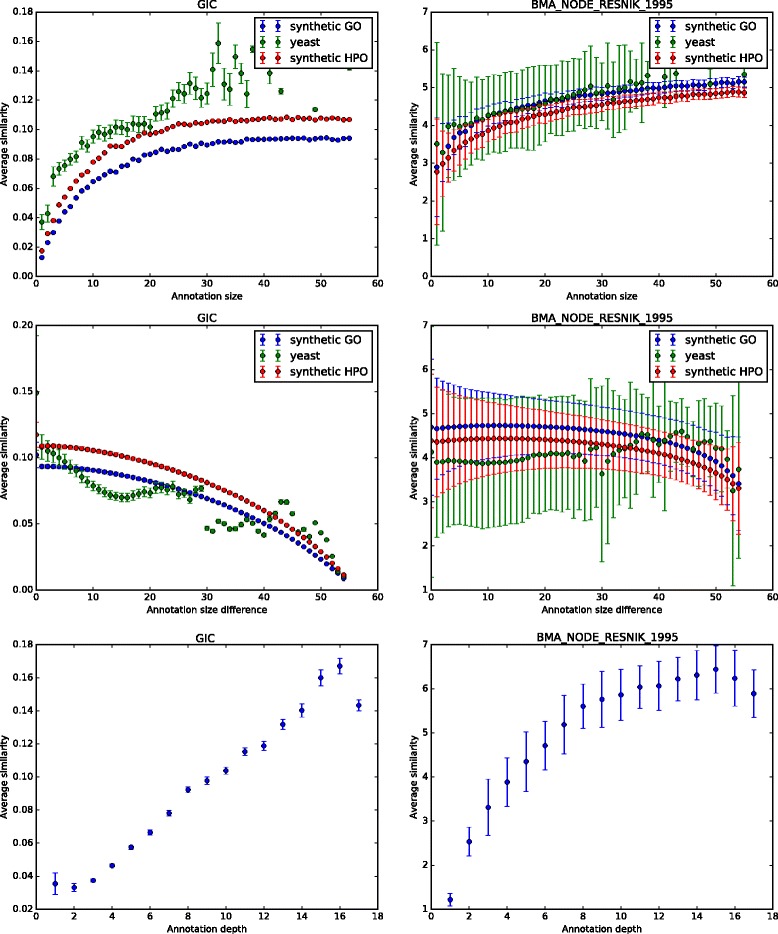
The distribution of similarity values as a function of the annotation size (*top*), annotation size difference (*middle*) and annotation class depth (*bottom*) for Resnik’s measure (using the Best Match Average strategy) and the simGIC measure

To measure the effects of annotation size, we fix the number of annotations of entities in our test corpus, and compare those with a certain number of annotations to all other entities. As we have generated 100 entities for each of the 55 annotation sizes in our corpus, we obtain a distribution of 550,000 (100 × 5500) similarity values for each annotation size. In the resulting distribution of similarity values, we compute average (arithmetic mean) similarity and variance. To determine if, and how much, the similarity values increase with annotation size, we compute Spearman and Pearson correlation coefficients for each similarity measure. The results for a selected set of similarity measures are shown in Table 1, and for Resnik’s similarity measure [20] (with the Best Match Average strategy for combining pairwise measures) and the simGIC measure [1] in Fig 1. We find that, in general and across almost all similarity measures, similarity values increase with the number of annotations associated with an entity. The variance in the average similarities, however, either increases or decreases with the annotation size, depending on the similarity measure.

**Table 1:** Spearman and Pearson correlation coefficients between similarity value and absolute annotation size as well as between variance in similarity value and annotation size

Similarity measure	Spearman						Pearson					
	Yeast		Synthetic GO		Synthetic HPO		Yeast		Synthetic GO		Synthetic GO	
	Average	Variance	Average	Variance	Average	Variance	Average	Variance	Average	Variance	Average	Variance
GIC (Graph Information Content)	0.929780	0.251586	0.970924	–0.773449	0.953247	–0.980159	0.861348	0.117734	0.831167	–0.744321	0.802873	–0.958817
NTO (Normalized Term Overlap)	0.178345	–0.860012	0.248990	–0.976335	0.123304	–0.988240	–0.014072	–0.682683	–0.009088	–0.574883	–0.158914	–0.593458
UI (Union Intersection)	0.892631	0.298097	0.879582	–0.934921	0.729942	–0.995599	0.788675	0.030649	0.777515	–0.914405	0.736711	–0.935415
BMA with Jiang, Conrath 1997	0.960133	–0.892027	0.998773	–0.993506	0.999351	–0.996609	0.892576	–0.812184	0.895020	–0.629497	0.907974	–0.692269
BMA with Lin 1998	0.980519	–0.800362	0.998918	–0.994733	0.999134	–0.998052	0.925181	–0.772250	0.896497	–0.638574	0.917599	–0.677309
BMA with Resnik 1995	0.980519	–0.717457	0.998773	–0.994228	0.998918	–0.998124	0.939044	–0.703981	0.895107	–0.642652	0.917738	–0.675426
BMA with Schlicker 2006	0.980519	–0.800362	0.998918	–0.994733	0.999134	–0.998052	0.925181	–0.772250	0.896497	–0.638574	0.917599	–0.677309

To determine whether the results we obtain also hold for a real biological dataset, we further evaluated the semantic similarity between yeast proteins using a set of selected semantic similarity measures. We find that the results in our test corpus are also valid for the semantic similarly of yeast proteins. Figure 1 shows the average similarity of yeast proteins as a function of the annotation size for two semantic similarity measures.

For example, the protein YGR237C has only a single annotation, and the average similarly, using the simGIC measure, is 0.035 across the set of all yeast proteins. On the other hand, protein CDC28, a more richly annotated protein with 55 annotations, has as average similarly 0.142 (more than 4-fold increase). These results suggest that some entities have, on average and while comparing similarity to exactly the same set of entities, higher similarity, proportional to the number of annotations they have.

As our second experiment, we evaluate whether the difference in annotation size has an effect on the similarity measure. We follow the same strategy as in our first experiment: we have used the same datasets but measured the average similarities as function of absolute difference of compared entities. For the annotation sizes from 1 to 55 we get 55 groups of similarities with annotation size difference from 0 to 54, and for each group we computed average similarity and variance in similarity values. Furthermore, we computed Pearsson and Spearman correlation coefficients between annotation size difference and average similarities to determine the sensitivity of the similarity to annotation size difference. Figure 1 shows our results using synthetic data as well as functional annotations of yeast proteins for Resnik’s similarity measure (using the Best Match Average strategy) and the simGIC measure, and Table 2 summarizes the results. Full results are available as supplementary material. We find that for most measures, average similarity decreases as the difference in annotation size increases, while the variance in similarity values behaves differently depending on the similarity measure.

**Table 2:** Spearman and Pearson correlation coefficients between similarity value and difference in annotation size as well as between variance in similarity value and difference in annotation size

Similarity measure	Spearma						Pearson					
	Yeast		Synthetic GO		Synthetic HPO		Yeast		Synthetic GO		Synthetic GO	
	Average	Variance	Average	Variance	Average	Variance	Average	Variance	Average	Variance	Average	Variance
GIC (Graph Information Content)	–0.895310	–0.931818	–0.999928	–0.999784	–0.999784	–0.997835	–0.875583	–0.503795	–0.964250	–0.484246	–0.963553	–0.496135
NTO (Normalized Term Overlap)	0.901443	–0.233045	0.999784	0.961833	0.999784	0.959524	0.882986	–0.192168	0.990210	0.848649	0.993038	0.849263
UI (Union Intersection)	–0.909524	–0.924459	–1.000000	–0.658658	–1.000000	–0.518687	–0.906605	–0.596963	–0.963476	–0.547645	–0.963569	–0.508495
BMA with Jiang, Conrath 1997	0.283838	–0.925830	–0.902597	–0.521861	–0.891486	–0.770130	0.074788	–0.850654	–0.834208	–0.495874	–0.848264	–0.735985
BMA with Lin 1998	0.462843	–0.674892	–0.901587	–0.552237	–0.891126	–0.731530	0.303157	–0.707318	–0.836486	–0.517670	–0.852998	–0.693744
BMA with Resnik 1995	0.578211	–0.579149	–0.901587	–0.537807	–0.891126	–0.699856	0.442458	–0.487544	–0.835991	–0.507179	–0.854007	–0.670199
BMA with Schlicker 2006	0.462843	–0.674892	–0.901587	–0.552237	–0.891126	–0.731530	0.303157	–0.707318	-0.836486	–0.517670	–0.852998	–0.693744

In our third experiment, we evaluate whether the depth of the annotation classes has an effect on the similarity measure. We use our fourth dataset which we randomly generated based on the depth of classes in the GO. The maximum depth in GO is 17, and we generate 17 groups of random annotations. We then compute the average similarity of the synthetic entities within one group to all the other groups, and report Pearsson and Spearman correlation coefficients between annotation class depth and average similarities to determine the sensitivity of the similarity to annotation class depth. Figure 1 shows our results using synthetic data as well as functional annotations of yeast proteins for Resnik’s similarity measure (using the Best Match Average strategy) and the simGIC measure, and Table 2 summarizes the results. We find that for most measures, average similarity increases with the depth of the annotations, i.e., the more specific a class is the higher the average similarity to other classes.

### A classification of similarity measures

Our finding allows us to broadly group semantic similarity measures into groups depending on their sensitivity to annotation size and difference in annotation size. We distinguish positive correlation (Pearsson correlation >0.5), no correlation (Pearsson correlation between −0.5 and 0.5), and negative correlation (Pearsson correlation <0.5), and classify the semantic similarity measures based on whether they are correlated with annotation size, difference in annotation size, and depth. Additional file 1: Table S1 provides a comprehensive summary of our results.

By far the largest group of similarity measures has a positive correlation between annotation size and similarity value, and a negative correlation between variance and annotation size. Popular similarity measures such as Resnik’s measure [20] with the Best Match Average combination strategy, and the simGIC similarity measure [23], fall in this group. A second group of similarity measures has no, or only small, correlation between annotation size and similarity values, and might therefore be better suited to compare entities with a large variance in annotation sizes. The Normalized Term Overlap (NTO) measure [24] falls into this group. Finally, a third group results in lower similarity values with increasing annotation size.

### Impact on data analysis

In order to test our results on an established biological use case involving computation of semantic similarity, we conducted an experiment by predicting protein-protein interactions using the similarity measures. Prediction of protein-protein interactions is often used to evaluate and test semantic similarity measures [8–10], but similar methods and underlying hypotheses are also used for candidate gene prioritization [25] in guilt-by-association approaches [12].

**Fig. 2:**
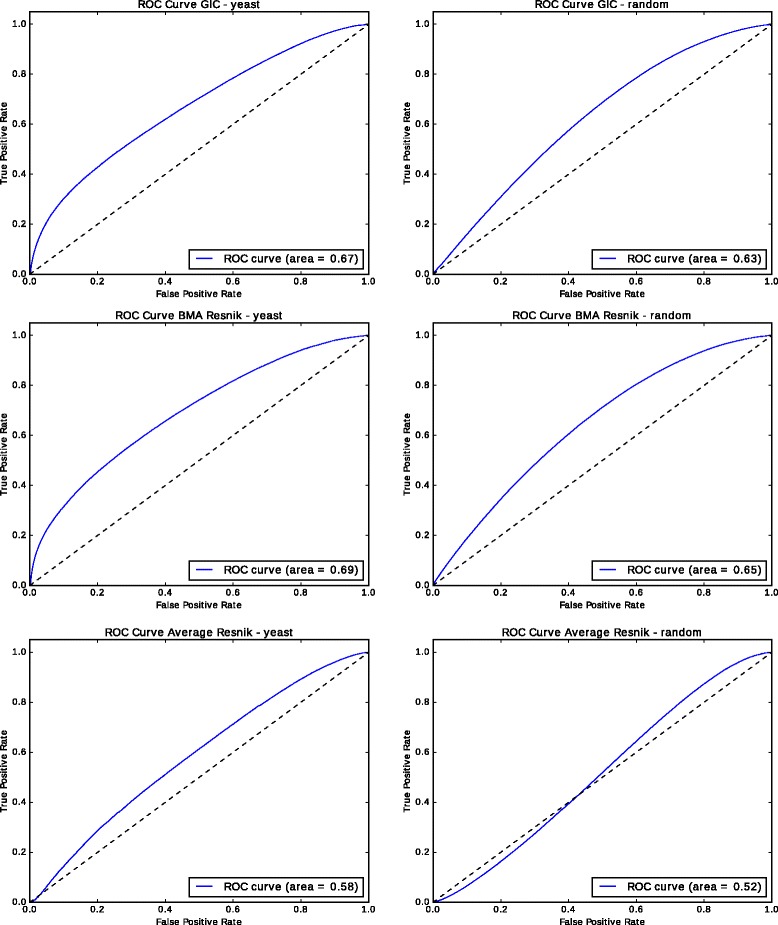
ROC Curves for protein-protein interaction prediction using random annotations and interaction data from BioGRID for yeast

**Fig. 3:**
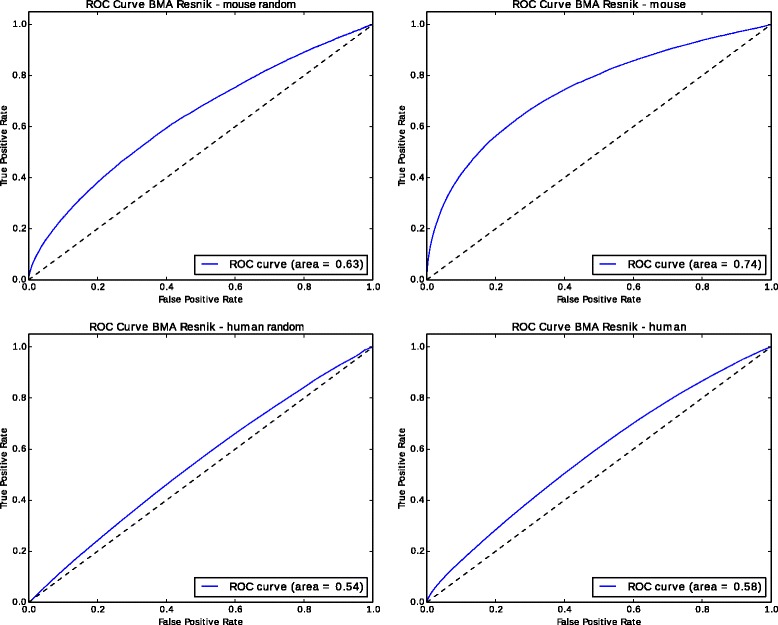
ROC Curves for protein-protein interaction prediction using random annotations and interaction data from BioGRID for mouse and human

We use our manually curated set of yeast gene annotations and then generated random GO annotations for each protein in this set while maintaining the annotation size fixed. Specifically, to generate a completely random annotation dataset, we replace each GO annotation of each protein in our yeast dataset by a random GO class. Thereby, the number of annotations for each protein remains constant, while the content of the annotation is replaced by a random GO class. We then compute pairwise semantic similarity between the proteins, once using the real annotations and additionally using the randomly generated annotations, and we use the resulting ranking as prediction of a protein-protein interaction. Using real protein-protein interactions from the BioGRID database [19], we compute the true positive rate and false positive rate of the predictions for each rank and plot the receiver operating characteristic (ROC) curves for both cases. The ROC curves are shown in Fig. 2 for simGIC and Resnik similarity measure. For example, for predicting PPIs using Resnik’s similarity measure and the BMA strategy, the area under the ROC curve (ROC AUC) using real biological annotations is 0.69, while the ROC AUC for random annotations is 0.65. Despite the complete randomization of the annotations, ROC AUC is significantly (*p*≤10^−6^, one-sided Wilcoxon signed rank test) better than random. We repeat this experiment with human and mouse PPIs and Resnik’s similarity measure (Fig. 3, and find that in each case, random annotations provide a predictive signal. For mouse PPIs, ROC AUC with random annotations is 0.63 while real GO annotations result in a ROC AUC of 0.74, and for human PPIs, ROC AUC with random annotations is 0.54 and 0.58 with real annotations. In both cases, the ROC curves are significantly better than random (*p*≤10^−6^, one-sided Wilcoxon signed rank test).

**Fig. 4:**
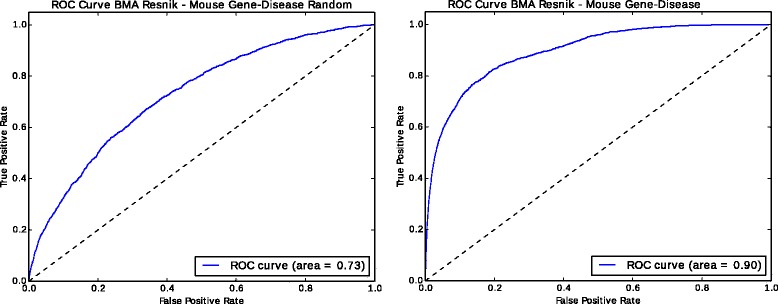
ROC Curves for gene-disease association prediction using PhenomeNet Ontology with mouse phenotype from MGI and OMIM disease phenotype annotations compared with random annotations

We further test if this phenomenon also holds for other applications of semantic similarity, in particular disease gene prioritization through phenotype similarity. For this purpose, we use the PhenomeNET systems [6, 26] and compare the semantic similarity associated with loss of function mouse models and human disease phenotypes. Using real annotations, ROC AUC is 0.90, while the ROC AUC for random phenotype annotations is 0.73 (Fig. 4), demonstrating that the phenomenon also holds for other use cases besides predicting PPIs.

The good performance in predicting PPIs in the absence of biological information is rather surprising. We hypothesized that well-studied proteins generally have more known functions and more known interactions, and also that genes involved in several diseases have more phenotype annotations. The Pearson correlation coefficient between the number of interactions and the number of functions in our yeast dataset is 0.34, in the human dataset 0.23, and 0.36 in the mouse PPI dataset. Similarly, in our dataset of gene–disease associations, there is a correlation between the number of phenotype annotations and the number of gene–disease associations (0.42 Pearson correlation coefficient). While the correlations are relatively small, there is nevertheless a bias that is confirmed by selecting a similarity measure that follows the same bias. We tested whether the same phenomenon occurs with another similarity measure that is not sensitive to the annotation size or difference in annotation size. Using Resnik’s measure with the Average strategy for combining the similarity values, we obtain a ROC AUC of 0.52 when predicting yeast PPIs. Although this ROC AUC is still significantly better than random (*p*≤10^−6^, one-sided Wilcoxon signed rank test), the effect is much lower compared to other measures.

In the context of gene networks, prior research has shown that the amount of functional annotation and network connectivity may result in biased results for certain types of analyses, leading the authors to conclude that the “guilt by association” principle holds only in exceptional cases [12]. Our analysis suggests that similar biases may be introduced in applications of semantic similarity measures such that heavily annotated entities will have, on average and without the presence of any biological relation between entities, a higher similarity to other entities than entities with only few annotations. A similar but inverse effect exists for differences in annotation size. Consequently, comparing entities with many annotations (e.g., well-studied gene products or diseases) to entities with few annotations (e.g., novel or not well-studied gene products) will result, on average, in the lowest similarity values, while comparing well-studied entities to other well-studied entities (both with high annotation size and no or only small differences in annotation size) will result in higher average similarity for most similarity measures even in the absence of any biological relation.

## Conclusions

We find that the annotation size of entities clearly plays a role when comparing entities through measures of semantic similarity, and additionally that the difference in annotation size also plays a role. This has an impact on the interpretation of semantic similarity values in several applications that use semantic similarity as a proxy for biological similarity, and the applications include prioritizing candidate genes [6], validating text mining results [27], or identifying interacting proteins [10]. Similarly to a previous study on protein-protein interaction networks [12], we demonstrate that the sensitivity of similarity measures to annotation size can lead to a bias when predicting protein-protein interactions. These results should be taken into account when interpreting semantic similarity values.

In the future, methods need to be identified to correct for the effects of annotation size and difference in annotation size. Adding richer axioms to ontologies or employing similarity measures that can utilize axioms such as disjointness between classes [28] does not on its own suffice to remove the bias we identify, mainly because the relation between annotated entities (genes or gene products) and the classes in the ontologies does not consider disjointness axioms. It is very common for a gene product to be annotated to two disjoint GO classes, because one gene product may be involved in multiple functions (such as “vocalization behavior” and “transcription factor activity”) since gene products are not instances of GO classes but rather are related by a *has function* relation (or similar) to some instance of the GO class. A possible approach could be to rely on the exact distribution of similarity values for individual entities [29] and use a statistical tests to determine the significance of an observed similarity value. An alternative strategy could rely on expected similarity values based on the distribution of annotations in the corpus and the structure of the ontology and adjusting similarity values accordingly so that only increase over expected similarity values are taken into consideration.

## Additional file

Additional file 1 Supplementary Table. (PDF 17 kb)

## Abbreviations

AUC: Area under curve
BMA: Best match average
GO: Gene ontology
HPO: Human phenotype ontology
NTO: Normalized term overlap
PPI: Protein-protein interaction
ROC: Receiver operating characteristic
SML: Semantic measures library
