# Supplementary material for: Evaluating the effect of annotation size on measures of semantic similarity
Source: J Biomed Semantics. 2017 Feb 13;8:7. doi: 10.1186/s13326-017-0119-z (PMC5307803; doi:10.1186/s13326-017-0119-z)
Supplement: Additional file 1 — Supplementary Table. (PDF 17 kb) [file 13326_2017_119_MOESM1_ESM.pdf]

Supplementary Table

Table 1 Similarity measures classified by Pearson correlation coefficients between similarity value and annotation size

Similarity Measure	Pearsson correlation coefficient
**Positive correlation**	
SIM LEE 2004	0.998575
BMM NODE TVERSKY IC PROP RATIO MODEL	0.997745
BMM EDGE SLIMANI 2006	0.997702
BMM NODE FEATURE TVERSKY RATIO MODEL	0.997235
BMM NODE JACCARD IC	0.997220
BMM NODE SCHLICKER JACCARD	0.997220
BMM EDGE STOJANOVIC 2001	0.997102
BMM NODE LIN 1998 GraSM	0.997060
BMM NODE RESNIK 1995 GraSM	0.996722
BMM NODE SIM IC 2010	0.996694
BMM NODE JIANG CONRATH 1997 NORM	0.996427
BMM NODE LIN 1998	0.996339
BMM NODE SCHLICKER 2006	0.996339
BMM NODE SCHLICKER TVERSKY	0.996339
BMM NODE TVERSKY IC RATIO MODEL	0.996339
BMM NODE MAZANDU 2012	0.995958
BMM NODE RESNIK 1995	0.995892
BMM NODE JACCARD 3W IC	0.992229
BMM NODE SCHLICKER 3WJACCARD	0.992229
BMM NODE FEATURE TVERSKY CONTRAST MODEL	0.991930
BMM NODE RESNIK 1995 ANCESTORS	0.991615
MAX NODE TVERSKY IC PROP CONTRAST MODEL	0.989585
KNAPPE 2004	0.977611
MARYLAND BRIDGE 2003	0.977611
BMM NODE TVERSKY IC CONTRAST MODEL	0.971870
SIM TO	0.971744
AVERAGE NORMALIZED GOSIM NODE TVERSKY IC PROP CONTRAST MODEL	0.951308
MAX NORMALIZED GOSIM NODE TVERSKY IC PROP CONTRAST MODEL	0.951308
BMM SIM PAIRWISE RANDOM	0.951272
BMA NODE RESNIK 1995 ANCESTORS	0.949657
BMA NODE TVERSKY IC PROP RATIO MODEL	0.945567
MAX NODE RESNIK 1995 ANCESTORS	0.939981
BMA EDGE SLIMANI 2006	0.938373
AVERAGE NORMALIZED GOSIM NODE TVERSKY IC CONTRAST MODEL	0.931502
MAX NORMALIZED GOSIM NODE TVERSKY IC CONTRAST MODEL	0.931502
MAX NODE TVERSKY IC CONTRAST MODEL	0.931449
BMA NODE JACCARD IC	0.923770
BMA NODE SCHLICKER JACCARD	0.923770
BMA EDGE STOJANOVIC 2001	0.910018
BMA NODE FEATURE TVERSKY RATIO MODEL	0.907918
BMA NODE SIM IC 2010	0.907486
MAX NORMALIZED GOSIM NODE RESNIK 1995 ANCESTORS	0.907462
BMA NODE LIN 1998 GraSM	0.898757
BMA NODE RESNIK 1995 GraSM	0.897208
AVERAGE NORMALIZED GOSIM NODE TVERSKY IC PROP RATIO MODEL	0.896725
MAX NORMALIZED GOSIM NODE TVERSKY IC PROP RATIO MODEL	0.896725
MAX NODE TVERSKY IC PROP RATIO MODEL	0.896725
BMA NODE LIN 1998	0.896497
BMA NODE SCHLICKER 2006	0.896497
BMA NODE SCHLICKER TVERSKY	0.896497
BMA NODE TVERSKY IC RATIO MODEL	0.896497

BMA NODE MAZANDU 2012	0.895292
BMA NODE RESNIK 1995	0.895107
BMA NODE JIANG CONRATH 1997 NORM	0.895020
MAX NODE FEATURE TVERSKY CONTRAST MODEL	0.889790
AVERAGE NORMALIZED GOSIM NODE JACCARD IC	0.885099
AVERAGE NORMALIZED GOSIM NODE SCHLICKER JACCARD	0.885099
MAX NORMALIZED GOSIM NODE JACCARD IC	0.885099
MAX NORMALIZED GOSIM NODE SCHLICKER JACCARD	0.885099
MAX NODE JACCARD IC	0.885099
MAX NODE SCHLICKER JACCARD	0.885099
SIM LP	0.884267
AVERAGE NORMALIZED GOSIM EDGE STOJANOVIC 2001	0.874551
MAX NORMALIZED GOSIM EDGE STOJANOVIC 2001	0.874551
MAX EDGE STOJANOVIC 2001	0.874551
MAX EDGE SLIMANI 2006	0.873669
AVERAGE NORMALIZED GOSIM EDGE SLIMANI 2006	0.873625
MAX NORMALIZED GOSIM EDGE SLIMANI 2006	0.873625
BMA NODE FEATURE TVERSKY CONTRAST MODEL	0.872939
AVERAGE NORMALIZED GOSIM NODE FEATURE TVERSKY RATIO MODEL	0.864680
MAX NORMALIZED GOSIM NODE FEATURE TVERSKY RATIO MODEL	0.864680
MAX NODE FEATURE TVERSKY RATIO MODEL	0.864680
BADER 2003	0.861838
AVERAGE NORMALIZED GOSIM NODE RESNIK 1995 GraSM	0.861081
MAX NORMALIZED GOSIM NODE RESNIK 1995 GraSM	0.861081
BMA NODE JACCARD 3W IC	0.859515
BMA NODE SCHLICKER 3WJACCARD	0.859515
AVERAGE NORMALIZED GOSIM NODE LIN 1998 GraSM	0.859124
MAX NORMALIZED GOSIM NODE LIN 1998 GraSM	0.859124
MAX NODE LIN 1998 GraSM	0.859124
MAX NODE RESNIK 1995 GraSM	0.859105
AVERAGE NORMALIZED GOSIM NODE SIM IC 2010	0.847827
MAX NORMALIZED GOSIM NODE SIM IC 2010	0.847827
MAX NODE SIM IC 2010	0.847629
AVERAGE NORMALIZED GOSIM NODE RESNIK 1995	0.846959
MAX NORMALIZED GOSIM NODE RESNIK 1995	0.846959
AVERAGE NORMALIZED GOSIM NODE JIANG CONRATH 1997 NORM	0.845821
MAX NORMALIZED GOSIM NODE JIANG CONRATH 1997 NORM	0.845821
MAX NODE JIANG CONRATH 1997 NORM	0.845821
AVERAGE NORMALIZED GOSIM NODE MAZANDU 2012	0.845348
MAX NORMALIZED GOSIM NODE MAZANDU 2012	0.845348
MAX NODE MAZANDU 2012	0.845348
AVERAGE NORMALIZED GOSIM NODE LIN 1998	0.844864
AVERAGE NORMALIZED GOSIM NODE SCHLICKER 2006	0.844864
AVERAGE NORMALIZED GOSIM NODE SCHLICKER TVERSKY	0.844864
AVERAGE NORMALIZED GOSIM NODE TVERSKY IC RATIO MODEL	0.844864
MAX NORMALIZED GOSIM NODE LIN 1998	0.844864
MAX NORMALIZED GOSIM NODE SCHLICKER 2006	0.844864
MAX NORMALIZED GOSIM NODE SCHLICKER TVERSKY	0.844864
MAX NORMALIZED GOSIM NODE TVERSKY IC RATIO MODEL	0.844864
MAX NODE LIN 1998	0.844864
MAX NODE SCHLICKER 2006	0.844864
MAX NODE SCHLICKER TVERSKY	0.844864
MAX NODE TVERSKY IC RATIO MODEL	0.844864
MAX NODE RESNIK 1995	0.844850
SIM GIC	0.831167
OCHIAI 1957	0.813173
AVERAGE NORMALIZED GOSIM NODE JACCARD 3W IC	0.801676
AVERAGE NORMALIZED GOSIM NODE SCHLICKER 3WJACCARD	0.801676

MAX NORMALIZED GOSIM NODE JACCARD 3W IC	0.801676
MAX NORMALIZED GOSIM NODE SCHLICKER 3WJACCARD	0.801676
MAX NODE JACCARD 3W IC	0.801676
MAX NODE SCHLICKER 3WJACCARD	0.801676
AVERAGE NORMALIZED GOSIM NODE FEATURE TVERSKY CONTRAST MODEL	0.792761
MAX NORMALIZED GOSIM NODE FEATURE TVERSKY CONTRAST MODEL	0.792761
BRAUN BLANQUET 1932	0.788073
SIM NTO MAX	0.788073
BATET 2010	0.787840
SOKAL SNEATH 1963	0.787228
JACCARD 1901	0.777515
SIM UI	0.777515
DICE 1945	0.760350
TVERSKY 1977	0.760350
BMA SIM PAIRWISE RANDOM	0.676415
KORBEL 2002	0.617517
BMM NODE TVERSKY IC PROP CONTRAST MODEL	0.616072
MAX SIM PAIRWISE RANDOM	0.599602
BMA NODE TVERSKY IC CONTRAST MODEL	0.595404
**No correlation**	
BMA NODE TVERSKY IC PROP CONTRAST MODEL	0.452011
BMA DIST NODE JIANG CONRATH 1997	0.413756
MAX DIST NODE JIANG CONRATH 1997	0.362606
MAX HYBRID RANWEZ 2006	0.247470
AVERAGE NODE FEATURE TVERSKY RATIO MODEL	0.211776
AVERAGE NORMALIZED GOSIM DIST NODE JIANG CONRATH 1997	0.198867
MAX NORMALIZED GOSIM DIST NODE JIANG CONRATH 1997	0.198867
BMM DIST NODE JIANG CONRATH 1997	0.195239
AVERAGE NODE TVERSKY IC PROP RATIO MODEL	0.194259
AVERAGE EDGE STOJANOVIC 2001	0.174854
AVERAGE EDGE SLIMANI 2006	0.163296
AVERAGE NODE FEATURE TVERSKY CONTRAST MODEL	0.156907
AVERAGE NODE JIANG CONRATH 1997 NORM	0.118293
AVERAGE NODE TVERSKY IC PROP CONTRAST MODEL	0.115284
AVERAGE NODE SIM IC 2010	0.049151
AVERAGE NODE LIN 1998	0.042000
AVERAGE NODE SCHLICKER 2006	0.042000
AVERAGE NODE SCHLICKER TVERSKY	0.042000
AVERAGE NODE TVERSKY IC RATIO MODEL	0.042000
AVERAGE NODE LIN 1998 GraSM	0.040206
AVERAGE NODE JACCARD 3W IC	0.039384
AVERAGE NODE SCHLICKER 3WJACCARD	0.039384
AVERAGE NODE TVERSKY IC CONTRAST MODEL	0.039143
AVERAGE NODE JACCARD IC	0.036728
AVERAGE NODE SCHLICKER JACCARD	0.036728
AVERAGE NODE MAZANDU 2012	0.032512
AVERAGE NODE RESNIK 1995	0.029337
AVERAGE NODE RESNIK 1995 GraSM	0.027080
AVERAGE NODE RESNIK 1995 ANCESTORS	0.026465
BMA HYBRID RANWEZ 2006	0.010811
SIMPSON 1960	-0.009088
SIM NTO	-0.009088
BMM HYBRID RANWEZ 2006	-0.050816
AVERAGE DIST NODE JIANG CONRATH 1997	-0.118293
AVERAGE HYBRID RANWEZ 2006	-0.121081
MAX NORMALIZED GOSIM SIM PAIRWISE RANDOM	-0.259764

AVERAGE NORMALIZED GOSIM SIM PAIRWISE RANDOM	-0.261069
AVERAGE SIM PAIRWISE RANDOM	-0.337290
MIN NODE TVERSKY IC PROP RATIO MODEL	-0.343210
MIN NODE TVERSKY IC CONTRAST MODEL	-0.362413
MIN NODE JIANG CONRATH 1997 NORM	-0.362606
MIN NODE RESNIK 1995 ANCESTORS	-0.374619
MIN EDGE SLIMANI 2006	-0.380637
MIN NODE SIM IC 2010	-0.426585
MIN NODE JACCARD IC	-0.433153
MIN NODE SCHLICKER JACCARD	-0.433153
MIN NODE LIN 1998	-0.444175
MIN NODE SCHLICKER 2006	-0.444175
MIN NODE SCHLICKER TVERSKY	-0.444175
MIN NODE TVERSKY IC RATIO MODEL	-0.444175
MIN NODE LIN 1998 GraSM	-0.444994
MIN NODE MAZANDU 2012	-0.445402
MIN NODE RESNIK 1995	-0.445658
MIN NODE RESNIK 1995 GraSM	-0.446474
MIN NODE JACCARD 3W IC	-0.454243
MIN NODE SCHLICKER 3WJACCARD	-0.454243
**Negative correlation**	
MIN SIM PAIRWISE RANDOM	-0.599758
MIN NODE FEATURE TVERSKY RATIO MODEL	-0.752219
MIN EDGE STOJANOVIC 2001	-0.786666
MIN HYBRID RANWEZ 2006	-0.800449
MIN DIST NODE JIANG CONRATH 1997	-0.845821
MIN NODE TVERSKY IC PROP CONTRAST MODEL	-0.884895
MIN NODE FEATURE TVERSKY CONTRAST MODEL	-0.910967
